# Involvement of NT3 and P75^NTR^ in photoreceptor degeneration following selective Müller cell ablation

**DOI:** 10.1186/1742-2094-10-137

**Published:** 2013-11-14

**Authors:** Weiyong Shen, Ling Zhu, So-Ra Lee, Sook H Chung, Mark C Gillies

**Affiliations:** 1 Save Sight Institute, the University of Sydney, 8 Macquarie Street, Sydney 2000, Australia

**Keywords:** Müller cell, Microglia, Neurotrophins, p75^NTR^, NT3, Retinal degeneration

## Abstract

**Background:**

Neurotrophins can regulate opposing functions that result in cell survival or apoptosis, depending on which form of the protein is secreted and which receptor and signaling pathway is activated. We have recently developed a transgenic model in which inducible and patchy Müller cell ablation leads to photoreceptor degeneration. This study aimed to examine the roles of mature neurotrophin-3 (NT3), pro-NT3 and p75 neurotrophin receptor (P75^NTR^) in photoreceptor degeneration in this model.

**Methods:**

Transgenic mice received tamoxifen to induce Müller cell ablation. Changes in the status of Müller and microglia cells as well as expression of mature NT3, pro-NT3 and P75^NTR^ were examined by immunohistochemistry and Western blot analysis. Recombinant mature NT3 and an antibody neutralizing 75^NTR^ were injected intravitreally 3 and 6 days after Müller cell ablation to examine their effects on photoreceptor degeneration and microglial activation.

**Results:**

We found that patchy loss of Müller cells was associated with activation of surviving Müller cells and microglial cells, concurrently with reduced expression of mature NT3 and upregulation of pro-NT3 and P75^NTR^. Intravitreal injection of mature NT3 and a neutralizing antibody to P75^NTR^, either alone or in combination, attenuated photoreceptor degeneration and the beneficial effect was associated with inhibition of microglial activation.

**Conclusions:**

Our data suggest that Müller cell ablation alters the balance between the protective and deleterious effects of mature NT3 and pro-NT3. Modulation of the neuroprotective action of mature NT3 and pro-apoptotic pro-NT3/P75^NTR^ signaling may represent a novel pharmacological strategy for photoreceptor protection in retinal disease.

## Background

Neurotrophins are a family of neurotrophic factors including at least four neurotrophins: nerve growth factor (NGF), brain-derived neurotrophic factor (BDNF), and neurotrophins (NT)-3 and-4 [1]. They are synthesized as precursors that can be either cleaved intracellularly or secreted uncleaved as pro-neurotrophins. Neurotrophins bind to two distinct types of receptors, the Trk receptor tyrosine kinase family and the p75 neurotrophin receptor (p75^NTR^) [1,2]. In the retina, NGF preferentially binds TrkA, which is almost exclusively expressed by ganglion cells. BDNF and NT4 preferentially bind TrkB, which is widely expressed by ganglion, horizontal, dopaminergic amacrine and Müller cells. NT3 preferentially binds TrkC that is expressed by photoreceptors and Müller cells. Trk receptors preferentially respond to mature neurotrophins promoting neuronal survival, differentiation and synaptic function [1,2]. The expression of p75^NTR^ is almost exclusively confined to Müller cells in the retina [2-4]. In contrast to mature neurotrophins, pro-neurotrophins exert their apoptotic effect *via* a receptor complex containing p75^NTR^ and sortilin [2,5]. Thus, neurotrophins can regulate opposing cellular functions that result in cell survival or apoptosis, depending on which form of the protein is secreted and which receptor and signaling pathway is activated.

Many different types of insult can potently induce pro-neutrophins and p75^NTR^. Accumulation of pro-NGF and upregulation of p75^NTR^ have been found to be positively correlated with accelerated retinal neurodegeneration in diabetes [6-8]. Upregulation of p75^NTR^ has been observed during light-induced photoreceptor degeneration [2], ocular hypertension [9], ischemic injury [10] and optic nerve axotomy [3,11]. Genetic ablation of p75^NTR^ or biochemical blockage of p75^NTR^ activation attenuates neuronal death induced by pro-neurotrophins [2,5]. Binding of pro-NGF to p75^NTR^ has been reported to induce robust expression of neurotoxic factors, suggesting that ligand activation of p75^NTR^ in Müller cells may activate neurotoxic pathways through a paracrine mechanism that negates the protective effect of mature neurotrophins [3,12]. Notably, previous studies indicate that NGF and BDNF can be secreted as pro-forms in the retina under pathological conditions [2,4,13,14]. However, the involvement of pathological pro-NT3/P75^NTR^ signaling in photoreceptor degeneration remains to be elucidated.

Progressive dysfunction and death of photoreceptors is the major cause of loss of vision in most retinal diseases. There is increasing evidence that Müller cells are important for photoreceptor health [15,16]. We recently generated an transgenic model using a portion of the regulatory region of the retinaldehyde binding protein 1 (Rlbp1) gene as a cell-specific promoter along with a CreER/Lox-P approach for inducible Müller cell-specific gene targeting [17]. These Rlbp1-CreER transgenic mice were crossed with Rosa-DTA176 mice, a transgenic line carrying an attenuated form of the diphtheria toxin fragment A (DTA176) gene, for Müller cell ablation following tamoxifen induction [17]. Selective Müller cell ablation in adult mice led to photoreceptor degeneration, blood-retinal barrier breakdown and deep retinal neovascularisation [17]. These changes are common, critical features of many retinal diseases such as macular telangiectasia [18-20], age-related macular degeneration [21,22], diabetic retinopathy [23,24] and ischemic retinopathy [25]. In this study, we have utilized this unique transgenic model to examine the roles of abnormal expression of mature NT3, pro-NT3 and P75^NTR^ in the photoreceptor degeneration after selective Müller cell ablation.

## Methods

### Conditional Müller cell ablation in transgenic mice

Animal studies were performed in accordance with the Association for Research in Vision and Ophthalmology statement and were approved by The University of Sydney Animal Ethics Committee. Rlbp1-CreER mice were crossed with Rosa-DTA176 mice to produce Rlbp-CreER-DTA176 transgenic mice, which were used for conditional, selective Müller cell ablation as we have previously described [17]. Animals were screened by PCR to identify those carrying both Rlbp1 and DTA176 genes. Selective Müller cell ablation in transgenic mice was induced by daily intraperitoneal injection of tamoxifen (TMX, 3 mg in 0.2 ml sunflower oil) for 4 consecutive days at 6–8 weeks of age [17]. Mice not carrying the Rlbp1 Müller cell-specific promoter but carrying the DTA176 gene were used as controls in this study.

### Cryosection and flat-mount immunohistochemistry (IHC)

Eyes were briefly fixed in 4% paraformaldehyde for 5 min, and then anterior segments were removed. After post-fixation in 4% paraformaldehyde for 1 h, eye cups were either transferred to PBS containing 30% sucrose and then embedded in optimal cutting temperature compound for cryosection IHC or placed in PBS for retinal flat-mount IHC. For cryosection IHC, frozen sections were blocked with 5% normal goat serum and incubated with an antibody (Ab) to glutamine synthetase (GS, mouse monoclonal, 1:100; Millipore no. MAB302), glial fibrillary acidic protein (GFAP, rabbit polyclonal, 1:250; Dako no. Z0334), P75^NTR^ (rabbit polyclonal, 1:250; a gift from Dr. Moses V. Chao, New York University, School of Medicine; no. 9651) and ionized calcium binding adaptor molecule 1 (Iba-1, rabbit polyclonal, 1:500, Wako no. 019–19741). Bound antibodies were detected with Alexa Fluor 488 or 594-conjugated goat or donkey secondary antibodies (1:1,000; Invitrogen).

For flat-mount staining, dissected eye cups were fixed in 4% paraformaldehyde for 1 h and then placed in PBS at +4°C overnight. On the next day, retinas were isolated, rinsed in PBS and permeabilized with 1% Triton-X-100 containing 5% normal goat serum blocking solution for 2 h. Retinas were incubated in 100 μl of solution containing peanut-agglutinin (PNA) conjugated with Alexa Fluor 488 or 594 (10 μg/ml, Invitrogen, no. L-21409 and L-32459) to label cone photoreceptor outer segments and an Ab against Iba1 (1:500, Wako no. 019–19741) for retinal microglia in 0.1 M PBS with 1% BSA and 0.5% Triton X-100 overnight at +4°C. Retinal whole-mounts were counterstained with Hoechst for 5 min before mounting onto slides for confocal laser scanning microscopy. Images were processed and analyzed using computer-based image analysis software to determine the percentage of PNA- or Iba-1 stained area per field of view as described previously [17,26]. In brief, a gradient detection algorithm was applied to the original digital image and binary thresholding performed on the gradient image by selecting its mean gray value as the threshold. This procedure allowed sufficient identification of the subject profiles to calculate the percentages of PNA- or Iba-1-stained area per field of view.

### Intravitreal injection of mature NT3 and an antibody against P75^NTR^

Intravitreal injection was performed using a 32-gauge needle attached to a Hamilton syringe as we have previously described [17]. Intravitreal injections were performed in transgenic mice 3 and 6 days after TMX-induced Müller cell ablation, with one eye receiving 2 μl of testing reagent and the other eye receiving 2 μl of BSS as a control in each mouse. Doses injected were: (1) NT3, 0.4 μg (R&D Systems, catalog no. 267N3/CF); (2) P75^NTR^ rabbit polyclonal Ab (no. 9651, 1:1 dilution, a gift from Dr. Moses V. Chao); (3) 0.4 μg NT3 + 1:1 dilution of P75 Ab. Injected eyes were enucleated 10 days after TMX-induced Müller cell ablation for retinal whole-mount staining to examine changes in cone photoreceptor outer segments and microglial activation as described above.

### Western blot

For Western blot, proteins were extracted from retinas and their concentrations determined by DC protein assay. Equal amounts of protein were subjected to SDS-polyacrylamide gel electrophoresis then transferred to a PVDF membrane for Western blot. Membranes were probed with antibodies to GS (mouse monoclonal, 1:1,000; Millipore, no. MAB302), GFAP (mouse monoclonal, 1:5,000; Neomarker, no. MS-280-P), P75^NTR^ (rabbit polyclonal, a gift from Dr. Moses V. Chao, no. 9651), NT3 (rabbit polyclonal, 1:1,000, Alomone Laboratory, no. ANT-003), pro-NT3 (rabbit polyclonal, 1:500, Alomone Laboratory, no. ANT-012) and rhodopsin (mouse monoclonal, 1:500, Millipore no. MAB5356), guanine nucleotide-binding protein subunit alpha-1 (GNAT1, rabbit polyclonal, 1:500, Santa Cruz no. sc-389) and Gα protein transducin (Gαt, mouse monoclonal, 1:2,000, BD Transduction Laboratories no. 610589). After incubation with secondary antibodies conjugated with horseradish peroxidase, protein bands were visualized using the G:Box BioImaging systems and quantified using the GeneTools image scanning and analysis package. Protein expression was normalized to α-/β-tubulin (rabbit polyclonal, 1:2,000; Cell Signaling no. 2148), which serves as a loading control.

### Statistics

Results are expressed as mean ± SEM. Data were analyzed using paired or un-paired *t*-test with a *p* value <0.05 accepted as statistically significant.

## Results

### Patchy loss of Müller cells was accompanied by activation of surviving Müller cells

**Figure 1:**
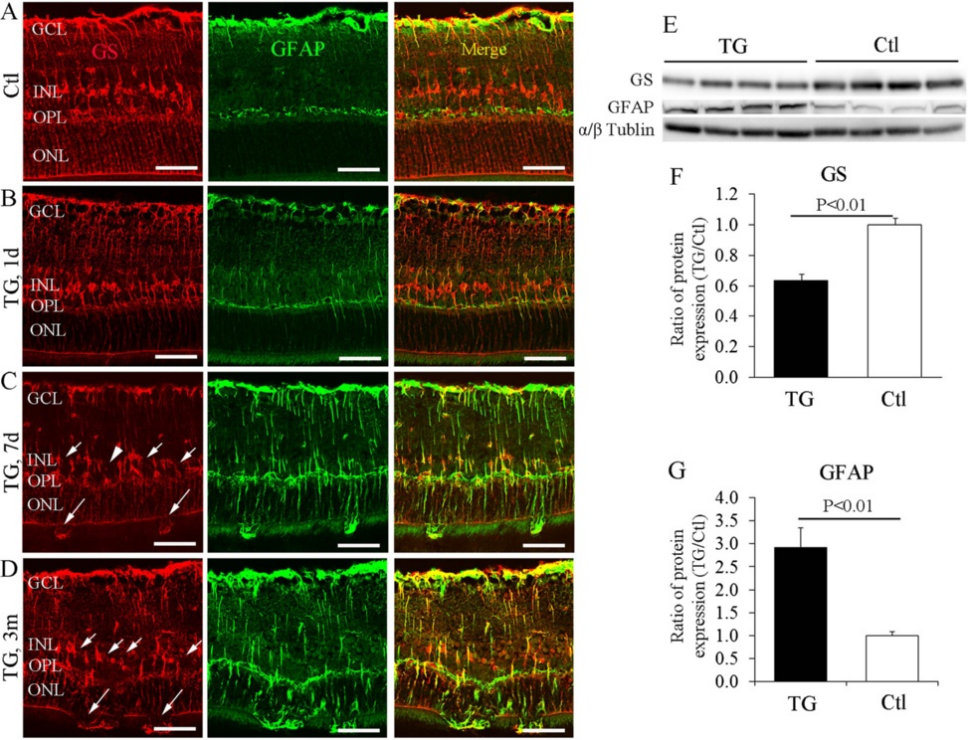
**Patchy loss of Müller cells was accompanied by reactive activation of surviving Müller cells after selective Müller cell ablation. (A**-**D)** Double-label immunostaining for GS (*red*, *right column*) and GFAP (*green*, *middle column*) in control (*Ctl*, **A**) and transgenic (*TG*, **B-D**) mice 1 day (1d), 7d and 3 months (3 m) after tamoxifen induction. Reactive activation of surviving Müller cells was observed as early as 1d **(B)** and persisted for at least 3 m **(D)** after Müller cell ablation in TG mice. *Small arrows* in **(C and D)** point to areas of Müller cell loss, and *large arrows* point to regions of broken outer limiting membrane (OLM). The *arrowhead* in **(C)** points to an area with Müller cell loss but the OLM at the corresponding region seemed intact. *GCL* = ganglion cell layer, *INL* = inner nuclear layer, *OPL* = outer plexiform layer, *ONL* = outer nuclear layer. **(E-G)** Western blots showed significant reduction of GS and upregulation of GFAP expression 7d after Müller cell ablation. *N* = 8 in each group. Scale bars: **A-D**, 50 μm.

We have previously shown patchy loss of Müller cells in Rlbp1-CreER-DTA176 transgenic mice, which can be observed as early as 1 day and becomes stable 14 days after induction with TMX [17]. We performed double-label IHC for GS and GFAP to examine the status of surviving Müller cells after selective Müller cell ablation (Figure 1A-D). In the control retina, strong GS immunoreactivity was observed across the neuroretina from the inner limiting membrane to the outer limiting membrane, with cell bodies localized to the inner nuclear layer, while GFAP expression was only observed in cells around the inner limiting membrane and in the outer plexiform layer (Figure 1A). In transgenic mice, reactive activation of surviving Müller cells was observed as early as 1 day after TMX treatment (Figure 1B), became more profound from 7 days (Figure 1C) and lasted for at least 3 months (Figure 1D) after TMX-induced Müller cell ablation. Western blot analysis demonstrated significant reduction in GS expression and upregulation of GFAP 7d after Müller cell ablation (Figure 1E and F). Thus, patchy loss of Müller cells induces reactive activation of surviving Müller cells in this model.

### Upregulation of P75^NTR^ in transgenic retinas after selective Müller cell ablation

**Figure 2:**
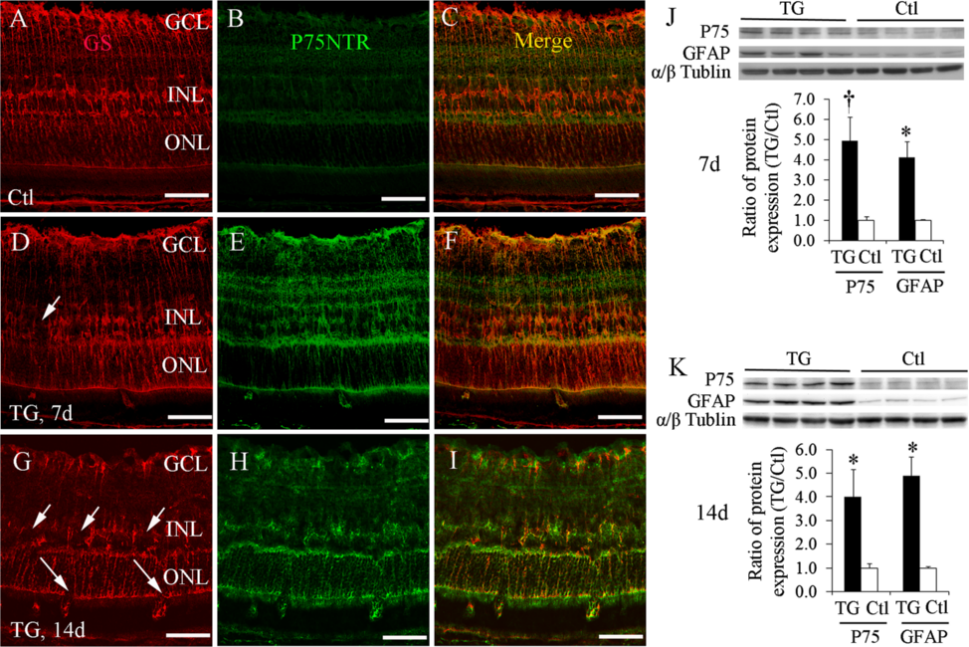
**Upregulation of P75**^**NTR **^**in transgenic retinas after Müller cell ablation. (A-I)** Double-label immunostaining for GS **(A, D and G)** and P75^NTR^**(B, E and H)** in control (*Ctl*, **A-C**) and transgenic (*TG*, **D-I**) mice 7d and 14d after tamoxifen treatment. The retina of control mice showed weak immunoreactivity of P75^NTR^**(A-C)**. TG mice showed upregulation of P75^NTR^ predominantly by surviving Müller cells 7d **(D-F)** and 14d **(G-I)** after TMX-induced Müller cell ablation. *Small arrows* in **(D and G)** point to regions of Müller cell loss, and *large arrows* in **(G)** indicate defects in the outer limiting membrane following Müller cell ablation. *GCL* = ganglion cell layer, *INL* = inner nuclear layer, *ONL* = outer nuclear layer. **(J and K)** Western blots showed significant upregulation of GFAP concomitantly with overexpression of P75^NTR^ 7d and 14d after Müller cell ablation in TG mice. ^†^*P* < 0.05 and ^*^P < 0.01, TG vs. control, *N* = 4–8 in each group. Scale bars: **A-I**, 50 μm.

As P75^NTR^ is predominantly expressed in Müller cells in the retina [2-4], we next examined whether these activated Müller cells overexpress P75^NTR^. Müller cells in the control retina expressed P75^NTR^ weakly (Figure 2A-C). By contrast, patchy loss of Müller cells was accompanied by strong immunostaining for P75^NTR^ in surviving Müller cells 7 and 14 days after Müller cell ablation (Figure 2D-I). Western blot results showed significant upregulation of P75^NTR^ and GFAP in transgenic mice 7 and 14 days after TMX treatment (Figure 2J and K). These results indicate that activated Müller cells overexpress P75^NTR^.

### Differential expression of mature NT3, pro-NT3, rod and cone phototransduction proteins

**Figure 3:**
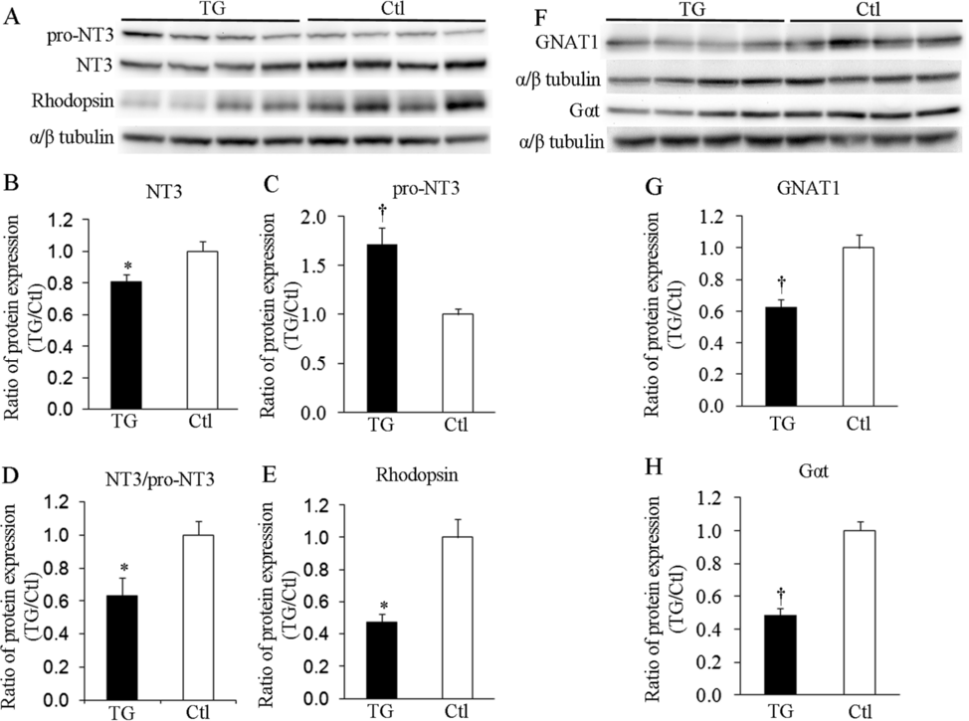
**Differential expression of mature NT3, pro-NT3, rod and cone phototransduction proteins 7d after Müller cell ablation. (A, F)** Western blots using antibodies to detect mature NT3, pro-NT3, rhodopsin, guanine nucleotide-binding protein subunit alpha-1 (GNAT1) and Gα protein transducin (Gαt). GNAT1 and Gαt are proteins essential for rod and cone phototransduction [27-29]. **(B-D)** Quantitative analysis of protein densitometry showed significant reduction in mature NT3 **(B)** and upregulation of pro-NT3 **(C)**, which resulted in a decreased ratio of NT3:pro-NT3 **(D)** 7d after Müller cell ablation. **(E, G, H)** The reduced expression of rhodopsin **(E)**, GNAT1 **(G)** and Gαt **(H)** in transgenic (TG) mice indicates that selective Müller cell ablation causes damage to both rod and cone photoreceptors. ^*^P < 0.05 and ^†^*P* < 0.01, TG vs. control (*Ctl*), *n* = 8/group in **(B, D and E)** and *n* = 11 − 13/group in **(C, G and H)**, respectively.

We next examined changes in mature NT3, pro-NT3, rod and cone phototransduction proteins after Müller cell ablation (Figure 3). Western blots were performed on retinal lysates using antibodies to detect mature NT3, pro-NT3, rhodopsin, guanine nucleotide-binding protein subunit alpha-1 (GNAT1) and Gα protein transducin (Gαt) 7 days after Müller cell ablation (Figure 3A and F). GNAT1 and Gαt are proteins essential for rod and cone phototransduction [27-29]. Quantitative analysis of protein densitometry revealed significant reduction in mature NT3 and upregulation of pro-NT3, which resulted in a decreased ratio of NT3:pro-NT3 (Figure 3A-D). The differential expression of mature NT3 and pro-NT3 was accompanied by significant reduction in rhodopsin (Figure 3E), GNAT1 (Figure 3G) and Gαt (Figure 3H) in TG mice, indicating that selective Müller cell ablation causes damage to both rod and cone photoreceptors.

### Activation of retinal microglial cells after selective Müller cell ablation

**Figure 4:**
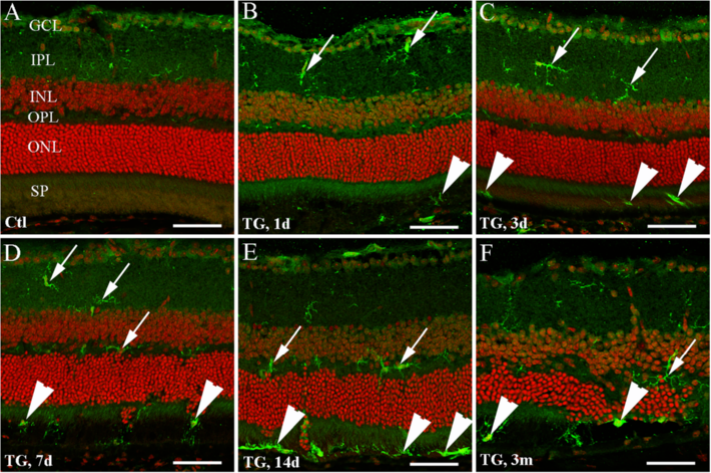
**Microglial activation after selective Müller cell ablation.** Immunohistochemistry was performed using an Ab against ionized calcium-binding adaptor molecule 1 (*Iba-1*, *green*) for microglia and nuclei were counterstained with Hoechst (*red*) to reveal retinal structure. **(A)** Control (*Ctl*) mice showing “resting” microglia cells in the ganglion cell layer (*GCL*), inner plexiform layer (*IPL*) and occasionally the outer plexiform layer (*OPL*), but not in the outer nuclear layer (*ONL*) or the subretinal space (*SP*). **(B-F)** Activation of microglia cells was observed in transgenic (*TG*) mice as early as 1 day (*1d*, **B**) after TMX-induced Müller cell ablation, as reflected by increased soma size and thickened cell processes in the inner retina (**B-F**, *arrows*). Microglia cells were observed in the subretinal space from 1d and frequently seen from 7d after TMX treatment (**B-F**, *arrowheads*). Note: protrusion of photoreceptor nuclei into the subretinal space in **(D-F)** and obvious thinning ONL in **(F)** 3 months (3 m) after TMX-induced Müller cell ablation. *Scale bars*: **A-F**, 50 μm.

Microglia and Müller cells are prominent participants in retinal responses to injury and diseases. There is evidence that the interaction between activated microglia and Müller cells can initiate a program of bidirectional microglia-Müller cell signaling that augments initial inflammatory responses to retinal injury [30]. We performed cryosection IHC using an Ab against Iba1 to examine microglial activation after Müller cell ablation (Figure 4). In the control retina, resting microglia cells were observed in the ganglion cell layer, inner plexiform layer and, occasionally, in the outer plexiform layer (Figure 4A). These resting microglia cells showed small somas with thin and ramified cells processes. They were predominantly confined to the inner retina but were found in neither the outer nuclear layer nor in the subretinal space (Figure 4A). Activation of microglia cells was observed in transgenic mice as early as 1 day after TMX-induced Müller cell ablation, as evidenced by expansion of their soma size and thickening of their cell processes (Figure 4B-F, arrows). Activated microglia cells were observed in the subretinal space from 1 day and frequently observed from 7 days after induced Müller cell ablation (Figure 4B-F, arrowheads).

**Figure 5:**
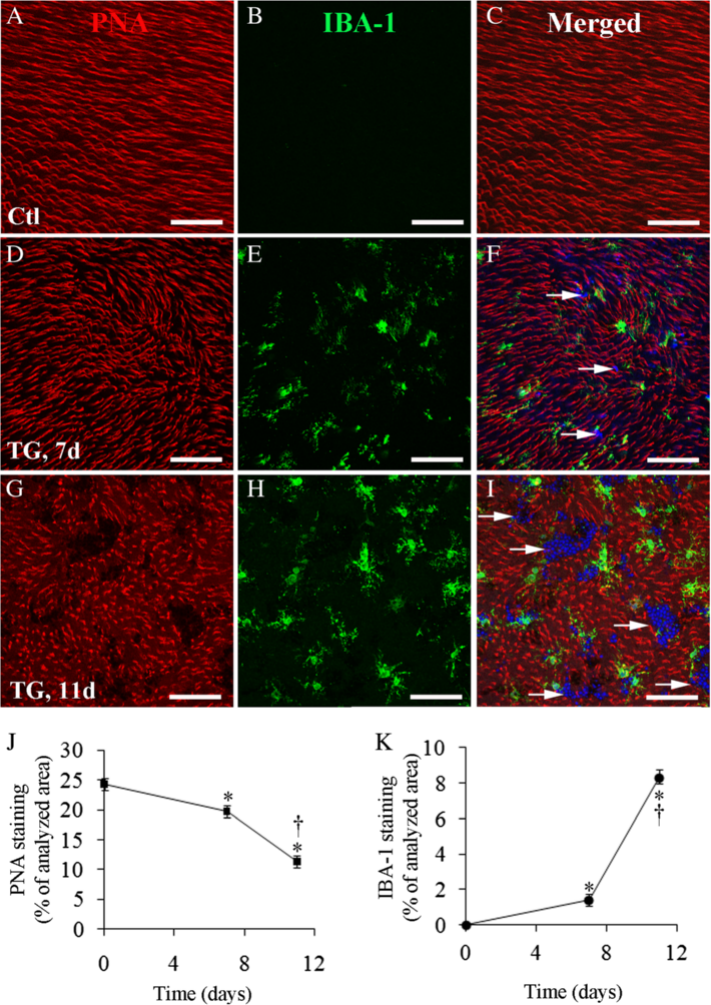
**Mapping cone photoreceptor outer segment loss with infiltration of activated microglial cells after Müller cell ablation. (A-I)** Double labeling using peanut-agglutinin (PNA)-conjugated with Alexa Fluor-594 for cone photoreceptor outer segments **(A, D and G**, *red*) and an Ab against ionized calcium binding adaptor molecule 1 (*IBA-1*) for microglia (**B, E and H**, *green*) on retinal whole mounts. **(A-C)** Images from a control (*Ctl*) retina. **(D-I)** Images from transgenic (*TG*) mice 7 and 11 days after tamoxifen-induced Müller cell ablation. **(C, F and I)** Merged images. Arrows in **(F and I)** point to areas of photoreceptor degeneration where degenerate cell bodies with nuclei stained blue protrude into the subretinal space. **(J and K)** Quantitative analysis of changes in PNA-labeled cone photoreceptor outer segments **(J)** and infiltration of activated microglial cells **(K)** 7 and 11 days after Müller cell ablation. ^*^P < 0.01, TG vs. Ctl and ^†^*P* < 0.01, 11d vs. 7d; unpaired *t*-test; *n* = 12–14 at each time point. Scale bars in **A-F**: 100 μm.

We further conducted double-label immunostaining on retinal whole mounts to map microglial activation with cone photoreceptor damage. Iba1 Ab was used to identify microglial cells, and lectin PNA was used to label cone photoreceptor outer segments (Figure 5). The control retina showed dense staining for PNA but lack of microglial infiltration at the level of photoreceptor outer segments (Figure 5A-C). In TG mice, most microglia retained slender cell processes, and PNA-staining showed limited loss of cone photoreceptor outer segments with only a few photoreceptor nuclei protruding into the subretinal space 7 days after induced Müller cell ablation (Figure 5D-F). Pronounced activation of microglial cells was evidenced by expansion of their soma size, which was clearly observed 11 days after Muller cell ablation (Figure 5G-I). Most microglia 11 days after Muller cell ablation (Figure 5H) had larger soma than at day 7 (Figure 5E), which is reflected by the increased area of microglial staining at day 11 compared with day 7 (Figure 5K) when the number of microglia seemed similar at both time points (Figure 5E and H). This was associated with more loss of photoreceptor outer segments at day 11 than at day 7 (Figure 5J).

### Photoreceptor protection after intravitreal injection of mature NT3 and P75^NTR^ Ab

**Figure 6:**
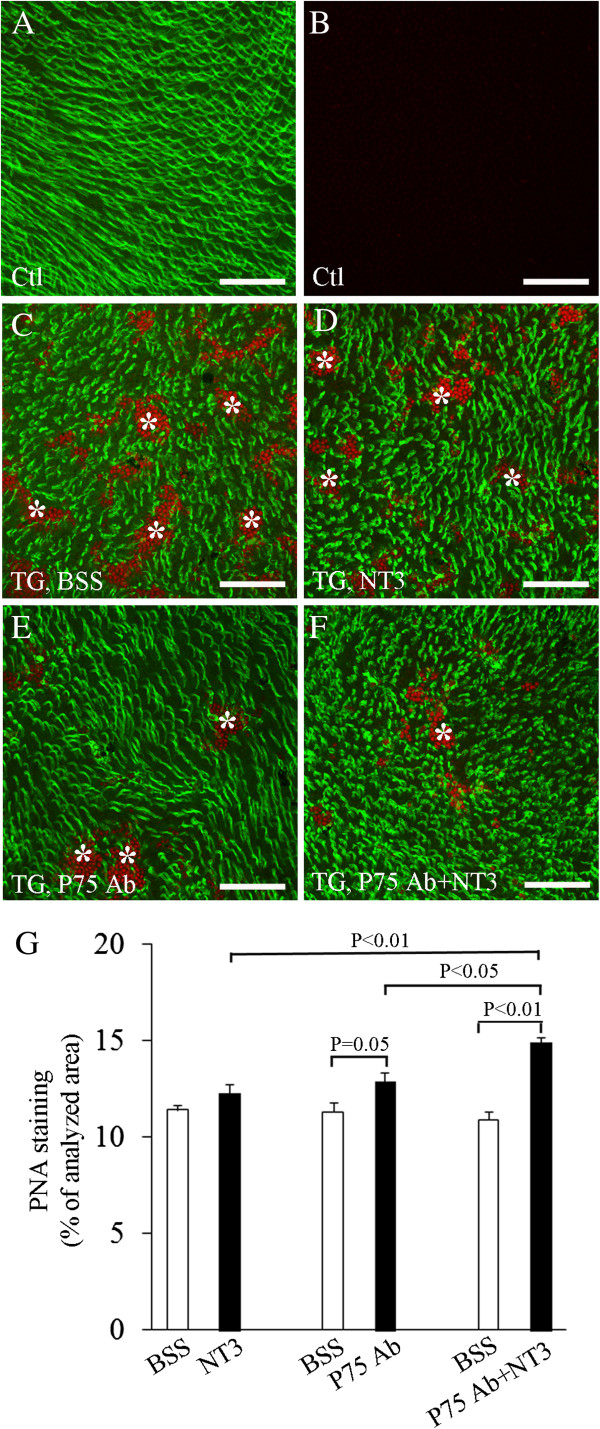
**Intravitreal injection of recombinant NT3 and blockage of P75**^**NTR **^**protected photoreceptor degeneration.** Intravitreal injection was performed in transgenic mice 3d and 6d after tamoxifen induced Müller cell ablation, with one eye receiving the testing reagent and the contralateral eye receiving balanced salt solution (BSS). Doses of injection: (1) NT3, 0.4 μg; (2) P75^NTR^ rabbit polyclonal Ab (1:1 dilution) and (3) 0.4 μg NT3 + 1:1 dilution of P75^NTR^ Ab. Eyes were enucleated 10d after TMX treatment for flat-mount staining using fluorescence-conjugated peanut-agglutinin (PNA). **(A and B)** PNA-labeled cone photoreceptor outer segments (*green*) and nuclear counterstaining in a control (*Ctl*) retina. **(C-F)** Images from transgenic (*TG*) mice showing changes in cone photoreceptor outer segments and protrusion of photoreceptor nuclei into the subretinal space (*red*, indicated by *asterisks*) in eyes receiving BSS **(C)**, NT3 **(D)**, P75^NTR^ Ab **(E)** and a combination of NT3 and P75^NTR^ Ab **(F)**. **(G)** Quantitative analysis of PNA staining showed that intravitreal injection of NT3 and P75 Ab protected the loss of cone photoreceptor outer segments, with a combined treatment more effective than either alone. *N* = 9 − 11/group. Scale bars in **A-F**: 100 μm.

Since selective Müller cell ablation resulted in reduced expression of mature NT3 and upregulation of pro-NT3 and P75^NTR^, we reasoned that intravitreal supplementation of exogenous mature NT3 and receptor blocking of P75^NTR^ might protect photoreceptors. We tested this hypothesis by performing intravitreal injections of recombinant mature NT3 and a neutralizing Ab to P75^NTR^ 3 and 6 days after TMX-induced Müller cell ablation. Changes in cone photoreceptor outer segments were analyzed 4 days after the last intravitreal injection (Figure 6). In control mice, intravitreal injection of NT3 in combination with P75^NTR^ Ab neither induced photoreceptor outer segment loss nor caused microglial infiltration in the subretinal space (Figure 6A and B). In TG mice, however, treatments with NT3 and P75^NTR^ Ab, either alone or in combination, reduced cone photoreceptor outer segment loss and photoreceptor nuclei protrusion when compared with eyes receiving BSS (Figure 6C-F). Quantitative analysis of changes in PNA-stained cone photoreceptor outer segments confirmed the beneficial effect of NT3 and P75^NTR^ Ab treatments on photoreceptor protection (Figure 6G).

### Inhibition of microglia after intravitreal injection of mature NT3 and P75^NTR^ Ab

**Figure 7:**
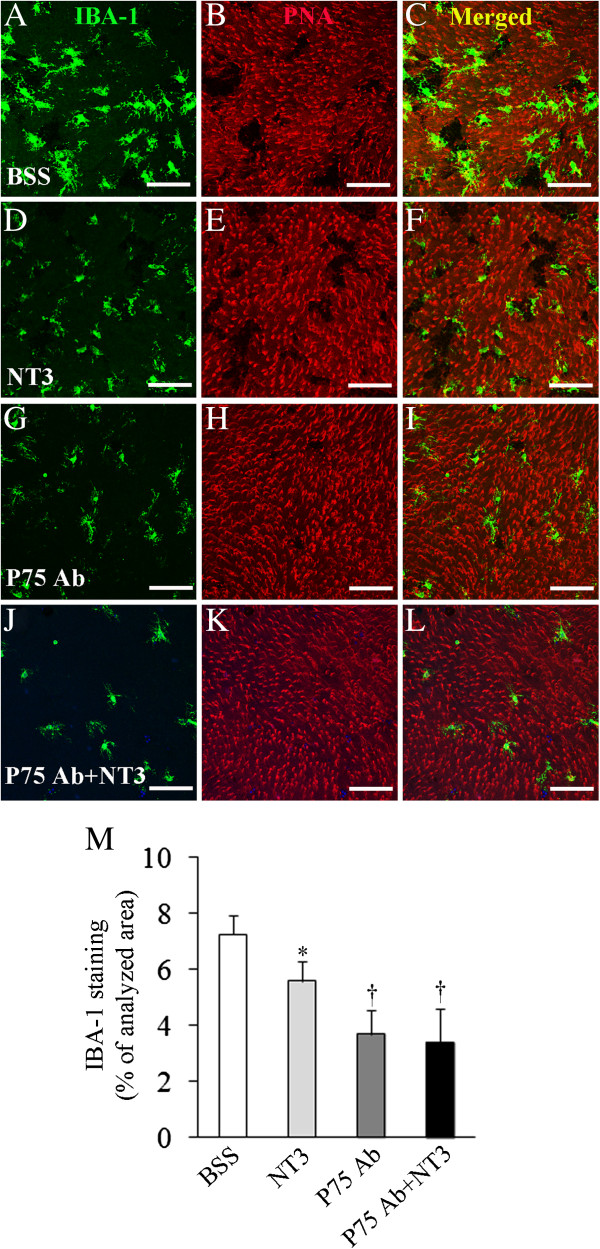
**Inhibition of microglial activation after intravitreal injection of recombinant mature NT3 and blockage of P75**^**NTR**^**.** Transgenic mice received twice intravitreal injections of NT3 (0.4 μg), P75^NTR^ rabbit polyclonal a polyclonal Ab to P75^NTR^ (1:1 dilution), a combination of both or balanced salt solution (*BSS*) 3d and 6d after tamoxifen (TMX)-induced Muller cell ablation. Eyes were enucleated 10d after TMX treatment for retinal flat-mount staining using an Ab against ionized calcium binding adaptor molecule 1 (*IBA-1*) for microglia (**A, D, G and J**, *green*) and peanut-agglutinin (*PNA*)-conjugated with Alexa Fluor-594 for cone photoreceptor outer segments (**B, E, H and K**, *red*). **(C, F, I and L)** Merged images. **(M)** Quantitative analysis of IBA-1 stained retinal whole mounts shows intravitreal injection of NT3 and P75^NTR^ blockage, either alone or in combination, protected photoreceptors concurrently with inhibition of microglial activation. ^*^P < 0.05 and ^†^*P* < 0.01, all vs. BSS injected group, *n* = 9 − 11/group. Scale bars in **A-L**: 100 μm.

We next examined the extent of microglial activation after intravitreal injection of mature NT3 and the P75^NTR^ neutralizing Ab. Consistent with our earlier findings that severe photoreceptor damage was accompanied by massive activation of microglia cells in this transgenic model (Figures 4 and 5), marked activation of microglia cells and loss of cone photoreceptor outer segments were observed in eyes receiving BSS (Figure 7A-C). In contrast, inhibition of microglial activation was observed in eyes receiving NT3, P75 Ab or both (Figure 7D, G and J). The extent of inhibition of microglial activation corresponded well with the extent of photoreceptor protection in eyes receiving of NT3, P75 Ab or the combined treatment (Figure 7F, I and L). Quantitative analysis of Iba-1 staining retinal whole mounts showed that intravitreal injection of NT3 and P75^NTR^ Ab, either alone or in combination, significantly inhibited microglial activation (Figure 7M).

## Discussion

We have demonstrated here that patchy loss of Müller cells induced photoreceptor degeneration accompanied by activation of surviving Müller cells and microglia in this model. Photoreceptor degeneration was concomitant with reduced expression of mature NT3 and upregulation of pro-NT3 and P75^NTR^. We then demonstrated that intravitreal injection of mature NT3 and a neutralizing Ab to P75^NTR^, either alone or in combination, attenuated photoreceptor degeneration concurrently with inhibition of microglial activation. Our results suggest that aberrant expression of NT3, pro-NT3 and P75^NTR^ is associated with photoreceptor degeneration and that treatment with mature NT3 together with inhibition of pro-NT3/P75^NTR^ signaling may represent a novel pharmacological strategy for protecting photoreceptors in retinal disease.

The primary role of Müller cells in the healthy retina is to maintain retinal homeostasis. The importance of Müller cells for photoreceptor health is indicated by the photoreceptor degeneration that developed after selective Müller cell ablation in our transgenic model [17]. We demonstrated that while TMX induction of DTA expression in Müller cells induced patches of Müller cell loss, surviving Müller cells were reactively activated. Reactive activation of Müller cells has been observed in retinal damage caused by every conceivable insult, including ischemia, trauma, degeneration, inflammation or neuronal excitotoxicity [31-35]. Müller cell activation is believed to be important for protection or repair of neurons in the early stages of neuronal damage [32-35]. However, activated Müller cells may overreact to the primary events and mediate secondary injury through generation of neurotoxic substances such as inflammatory mediators, free radicals, lipid peroxidation, increased levels of extracellular calcium and release of excitatory amino acids [3,12,15,36,37].

The role of p75^NTR^ as an apoptotic receptor is implicated in promoting apoptosis of neurons in the central nervous system following various injuries [2,38-40]. The involvement of p75^NTR^ in photoreceptor cell death has been previously reported in various animal models of retinal degenerations [2,4,41,42]. In the present study, we have particularly examined changes in P75^NTR^ expression 7 and 14 days after TMX treatment because these are the times at which the rate of photoreceptor apoptosis peaks in this model [17]. Double-label IHC showed strong immunoreactivity for P75^NTR^ in surviving Müller cells, and Western blot analysis showed significant upregulation of P75^NTR^ and GFAP at these two time points. These findings suggest that activated Müller cells overexpress P75^NTR^. There is evidence that activation of the pro-NGF/P75^NTR^ signaling contributes to retinal neuronal injury through a paracrine mechanism in the retina [3,12]. It has been reported that activation of the pro-NGF/P75^NTR^ signaling induced robust expression of neurotoxic substances such as tumor necrosis factor alpha and α2 macroglobulin [3,12]. Whether this also occurs in our transgenic model warrants further investigation.

Photoreceptor cell injury in animal models of retinal degenerations is often accompanied by microglial activation. We found activated microglial cells in the subretinal space as early as 1 and 3 days after TMX-induced Müller cell ablation. Marked activation of microglial cells was observed as photoreceptors were degenerating, and our further analysis revealed a close association between microglial infiltration in the subretinal space and loss of photoreceptor outer segments. This close spatial relationship between the activated microglial cells and photoreceptor damage in this study suggests that microglial cells might be involved in photoreceptor degeneration. Microglial cells are the resident tissue macrophages and primary immune cells of the central nervous system and retina. Under basal conditions, “resting” microglia demonstrate ramified morphologies and extend fine processes through nearby neural parenchyma. Resting microglial cells play key roles in housekeeping functions such as pruning excess or dysfunctional synapses [43,44], distributing supportive growth factors to active neurons [45] and regulating synaptic function [46,47]. However, activated microglial cells phagocytose cell debris and aggregated proteins while concurrently secreting toxic compounds during injury in the central nervous system [48]. Activated microglial cells are reported to secrete cytotoxic factors including free oxygen intermediates, proteases and excitatory amino acids, which may induce neuronal degeneration [48-50]. Microglial cells also secrete tumor necrosis factor-α [51,52], interleukin-1 [30,53,54] and the pro-forms of NGF, BDNF, NT3 and NT4 [4]. It has been reported that microglia-derived pro-NGF promotes photoreceptor cell death via activation of pro-NGF/P75^NTR^ signaling [4]. Recent studies indicate that activated microglia and Müller cells can influence each other to initiate a program of bidirectional microglia-Müller cell signaling, thereby contributing to the neuroinflammatory response and photoreceptor death [30,55]. Therefore, it is conceivable that activated Müller cells and microglia may contribute to photoreceptor degeneration via release of neurotoxic compounds in our transgenic mice. Our observations are consistent with a previous study that reported that accumulation of activated microglia in the outer retina occurred concurrently with the wave of photoreceptor degeneration in a mouse model of subretinal hemorrhage [56].

The photoreceptor degeneration after Müller cell ablation could be due to either reduced NT3/TrkC signaling or enhanced pro-neurotrophin/p75^NTR^ signaling or both [2,13,57]. It has been reported that Müller cells, RPE cells and ganglion cells express mature NT3 [58-61] and activated microglial cells express pro-NT3 [4]. As Muller cells are the major source of neurotrophic factors such as NT3 [60], basic fibroblast growth factor and ciliary neurotrophic factor [62-64], loss of neurotrophic support from Muller cells would affect the survival of photoreceptors. Our previous study showed that intravitreal supplementation of ciliary neurotrophic factor attenuates photoreceptor injury in this transgenic model [17]. In the present study, we found that activation of microglial and surviving Müller cells was concomitant with significant overexpression of pro-NT3 and P75^NTR^ and reduction in mature NT3 after Müller cell ablation. We hypothesized that supplementation with exogenous mature NT3 and inhibition of pro-neurotrophins/P75^NTR^ signaling would protect photoreceptors. We tested this hypothesis by intravitreal injections of recombinant mature NT3 and a neutralizing Ab against P75^NTR^. Quantitative analysis of PNA-stained cone photoreceptor outer segments showed intravitreal injection of NT3 and inhibition of P75^NTR^, either alone or in combination, attenuated photoreceptor degeneration. These results further confirm a potentially critical role of abnormal expression of NT3, pro-NT3 and P75^NTR^ in photoreceptor degeneration. Previous studies have shown that activation of pro-NGF/P75^NTR^ signaling contributes to retinal neuronal damage in animal models of glaucoma, retinal degenerations and diabetes [2-4,6,8,9,65]. A recent study reported that exogenous delivery of the NT-3 gene can be neuroprotective in an animal model of focal cerebral injury [66]. There is evidence that mature NT3 stimulates Müller cells to produce bFGF, and this effect can be blocked by a NT3 neutralizing Ab [2]. Dorrell et al. have reported that targeted delivery of NT4 to activated Müller cells protects retinas from neuronal degeneration in Vldlr^−/−^ mice [67]. To our knowledge, our study is the first to show direct evidence that overexpressions of pro-NT3 and P75^NTR^ as well as downregulation of mature NT3 are associated with photoreceptor degeneration and that regulation of pro-NT3/P75^NTR^ signaling is effective in photoreceptor protection.

We also found that intravitreal injections of NT3 and inhibition of P75^NTR^ resulted in reduced microglial activation when they were given individually or together. In eyes receiving BSS injection, massive migration of activated microglial cells was accompanied by loss of photoreceptor outer segments. A recent study indicated that migration of activated microglial cells into the subretinal space transforms the environment of the outer retina from an immune-privileged zone into a highly proinflammatory region, which, in turn, potentiates cellular apoptosis and photoreceptor degeneration [56]. We found that the degree of microglial inhibition appeared to map well with the extent of photoreceptor protection after intravitreal injection of mature NT3 and the P75^NTR^ Ab. Since microglial cells can be activated by cell debris and aggregated proteins released from damaged tissue, the inhibition of microglia that we observed after treatment with mature NT3 might be due to a protective effect of NT3 on photoreceptors that would have limited the extent of damage. For eyes receiving the P75^NTR^ neutralizing Ab, it is possible that blocking P75^NTR^ reduced the production of inflammatory cytokines, chemotactic cytokines and adhesion molecules from activated Müller cells, thus preventing microglial activation and protecting photoreceptors [3,12,30,56,68]. The combined treatment by NT3 and P75^NTR^ Ab is likely to result in dual effects on neuroprotection and microglial inhibition.

## Conclusions

We have shown that patchy loss of Müller cells induces photoreceptor degeneration, which is accompanied by activation of surviving Müller cells and microglia. These changes were associated with reduced expression of mature NT3 and upregulation of pro-NT3 and P75^NTR^. The ability of intravitreal treatment with NT3 and the P75^NTR^ neutralizing Ab to protect photoreceptors and inhibit microglial activation indicates that manipulation of neuronal-Müller cell-microglial interactions may be a novel strategy for neuroprotection in retinal disease.

## Abbreviations

BNDF: Brain-derived neurotrophic factor; DTA: Diphtheria toxin fragment A; Gαt: Gα protein transducin; GFAP: Glial fibrillary acidic protein; GNAT1: Guanine nucleotide-binding protein subunit alpha-1; GS: Glutamine synthetase; Iba-1: Ionized calcium binding adaptor molecule 1; IHC: Immunohistochemistry; NGF: Nerve growth factor; NT-3: Neurotrophin-3; P75NTR: p75 neurotrophin receptor; PNA: Peanut-agglutinin; Rlbp1: Retinaldehyde binding protein 1; TMX: Tamoxifen.

## Competing interests

The authors declare that they have no competing interests.

## Authors’ contributions

WS was involved in the study design, performed the experiments, analyzed the data and images, and wrote the manuscript. LZ contributed to genotyping and Western blot analysis. SL contributed to genotyping, IHC, Western blot and data analysis. SC assisted with in vivo experiments. MG was involved in the study design and writing the manuscript. All authors have read and approved the final version of the manuscript.
